# Massive Hemorrhage Following Spontaneous Mediastinal Inflammatory Myofibroblastic Tumor Rupture: A Case Report

**DOI:** 10.7759/cureus.45833

**Published:** 2023-09-23

**Authors:** Sophia B Bellegarde, Vanessa Gibson, Shahriyour Andaz, Lillian Huang, Eric Robinson, George Turi, Donald Tofuah, Chukwuyem Ekhator, Svetoslav Bardarov

**Affiliations:** 1 Pathology and Laboratory Medicine, American University of Antigua, St. John's, ATG; 2 Cardiothoracic Surgery, Mount Sinai South Nassau, Oceanside, USA; 3 Thoracic Surgery, Mount Sinai South Nassau, Oceanside, USA; 4 Surgery, Mount Sinai South Nassau, Oceanside, USA; 5 Cardiothoracic Surgery, American University of Antigua, St. John's, ATG; 6 Neuro-oncology, New York Institute of Technology College of Osteopathic Medicine, Old Westbury, USA; 7 Pathology and Laboratory Medicine, Richmond University Medical Center, Staten Island, USA

**Keywords:** pathology, thoracotomy procedure, hemothorax, inflammatory myofibroblastic tumor, hypertensive emergency, mediastinal mass

## Abstract

A 45-year-old male in a hypertensive emergency was admitted with complaints of frontal headache, progressive chest discomfort, shortness of breath, dysphagia, and right upper quadrant abdominal pain radiating across the epigastrium and to the back that increases in intensity with deep inspiration. He denied any history of abdominal pain, vomiting, dyspnea, nausea, and weight loss. A computed tomography (CT) scan of the chest showed a posterior mediastinal mass between the esophagus and descending aorta. A magnetic resonance imaging (MRI) scan revealed a non-enhancing posterior mediastinal mass possibly compressing both the esophagus and the airway. A 30-degree thoracoscope was inserted in the chest cavity revealing a large hemothorax from a possibly ruptured inflammatory myofibroblastic tumor (IMT) encompassing nearly the entire pleural space with both fresh and clotted blood. Two liters of fresh blood was removed via a right thoracotomy procedure. Once removed, a large fibrinous clot-filled mass was resected entirely and sent to pathology. Postoperative recovery was uneventful; dysphagia and shortness of breath resolved. The patient gradually resumed his regular diet.

## Introduction

Inflammatory myofibroblastic tumors (IMTs) are complexes of myofibroblastic spindle cells that demonstrate an inflammatory infiltrate. Though these rare lesions can arise from any tissues in the human body, their development in the mediastinal region is very uncommon. Histologically, IMTs exhibit diverse appearances, ranging from fibroblastic and myofibroblastic proliferation to an inflammatory infiltrate rich in plasma cells, lymphocytes, and eosinophils [1]. Diagnosis relies on clinical, radiological, histological, and molecular findings [2]. Differential diagnosis includes various malignancies, such as sarcomas, lymphomas, and spindle cell neoplasms, as well as inflammatory conditions [3-5]. The integration of these aspects ensures accurate diagnosis and appropriate management. We report a case of massive hemothorax resulting from a ruptured inflammatory myofibroblastic tumor incidentally uncovered arising from the patient's mediastinal mesenchymal tissue. Symptoms and imaging resembled the pathophysiology of an esophageal cyst wrapped around the esophagus. The final diagnosis was confirmed by surgical pathology post thoracotomy and specimen excisional biopsy.

## Case presentation

**Figure 1:**
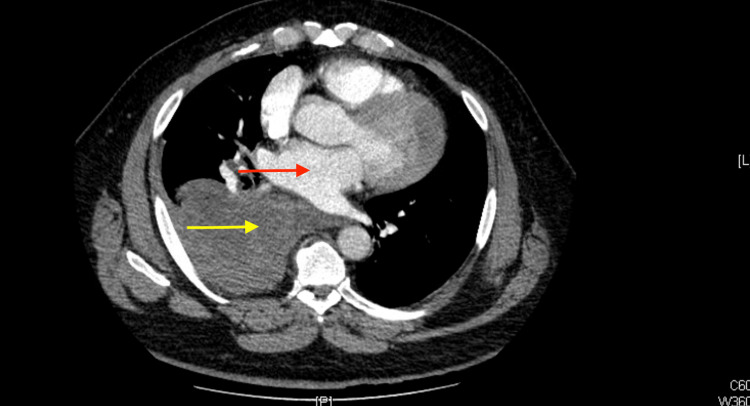
Computed tomography (CT) scan showing mediastinal mass (red arrow) with bleeding into the right pleural space (yellow arrow) resulting in hemothorax

A 45-year-old male presented in the emergency department in a hypertensive emergency (234/131) with complaints of frontal headache, progressive chest discomfort, shortness of breath, dysphagia, and right upper quadrant abdominal pain radiating across the epigastrium and to the back that increases in intensity with deep inspiration. He has a past medical history of asthma and obstructive sleep apnea on continuous positive airway pressure (CPAP) and has not been to a doctor's visit in over six years. A computed tomography (CT) scan of the chest was completed due to concerns of possibly ruptured posterior mediastinal mass versus mass effect with pleural effusion. A computed tomography (CT) scan in Figure 1 shows a mediastinal mass with bleeding into the right pleural space resulting in hemothorax with no evidence of pulmonary emboli seen. Magnetic resonance imaging (MRI) scan with IV contrast ordered also showed an 11.5 × 6.5 × 12.5 cm non-enhancing posterior mediastinal mass occupying the subcarinal region of the mediastinum displacing the esophagus and compressing the airway and possibly causing an interval enlargement of the hemothorax detected on imaging. Imaging demonstrates the detected mass lying between the aorta and the esophagus causing a plethora of symptoms in the patient.

**Figure 2:**
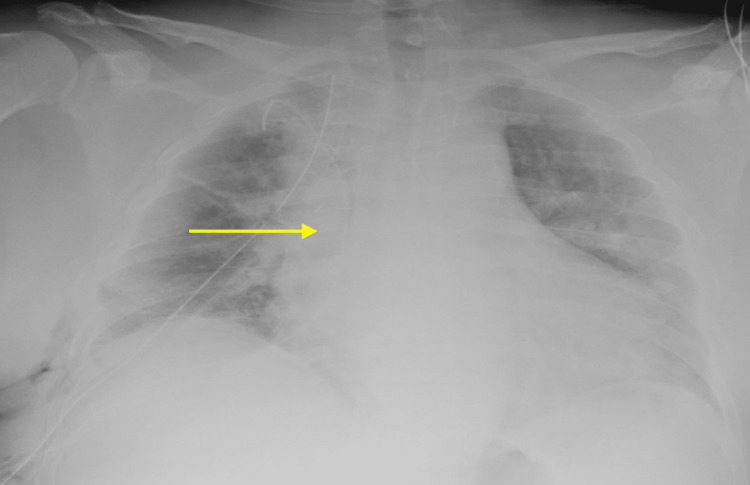
Chest X-ray (CXR) showing decreased right-sided consolidation and effusion (yellow arrow)

Upon the review of physical evaluation and imaging, surgical intervention was advised. Chest X-ray (CXR) result showed decreased right-sided consolidation and effusion compared with previous studies (Figure 2).

**Figure 3:**
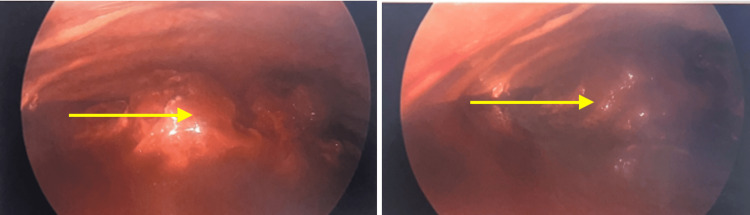
Thoracoscopy confirming a massive hemothorax (yellow arrow)

The thoracic team was consulted to proceed with elective thoracotomy, which confirmed a massive right hemothorax (Figure 3). A diagnostic right thoracotomy, converted to open thoracotomy approach, was undertaken by surgery. A double-lumen tracheal tube was placed. Thoracoscopy was performed to confirm the position of the endotracheal tube revealing an external compression of the right mainstem bronchus obliterating the lower airways. Invasive monitoring lines were placed. The patient was positioned in a left decubitus position, and a thoracostomy incision in the posterior axillary line at the eighth interstitial space was made. The chest cavity was entered. A 30-degree thoracoscope inserted in the chest cavity revealed a large hemothorax, which nearly obliterated the entire pleural space with both fresh and clotted blood present. Two liters of fresh blood was removed leaving behind a large organized fibrinous clot occupying the entire posterior mediastinum compressing the right lower lobe and the posterior aspect of the right upper lobe. The blood appeared to be coming from a ruptured portion of the pleura overlying the esophagus in the mid-thoracic space. The texture of the blood removed appeared mucinous and quite sticky. The azygos vein above the mass was ligated to prevent further bleeding.

**Figure 4:**
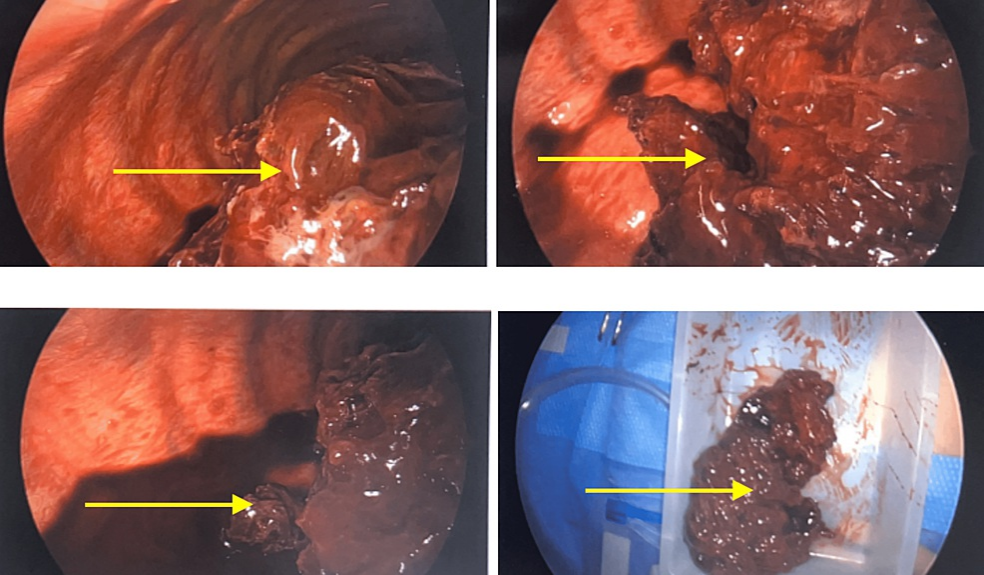
Right thoracoscopy revealing a large fibrinous necrotic mass (yellow arrow) measuring 11.5 × 6.5 × 12.5 cm, which was resected post hemothorax evacuation

**Figure 5:**
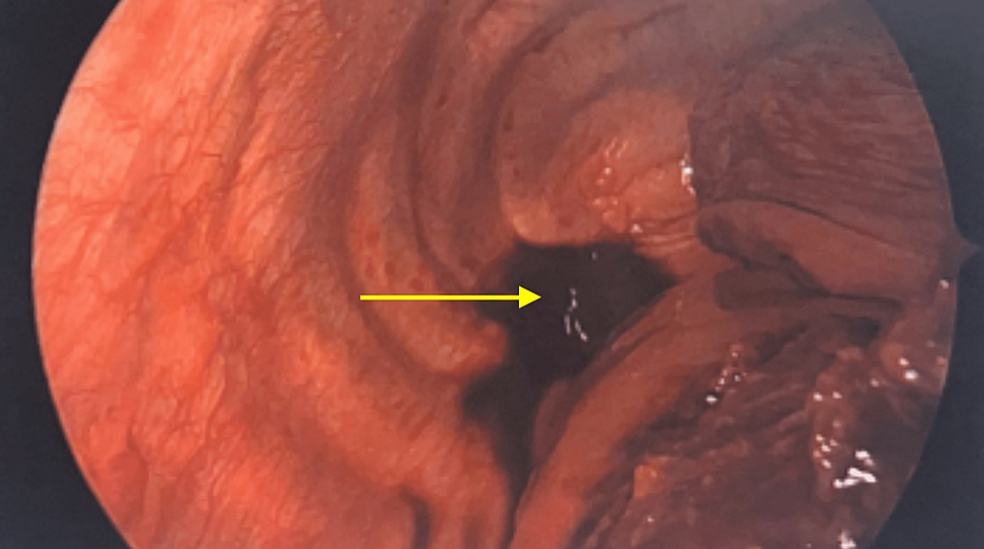
Right thoracic cavity (yellow arrow) post hemothorax evacuation

**Figure 6:**
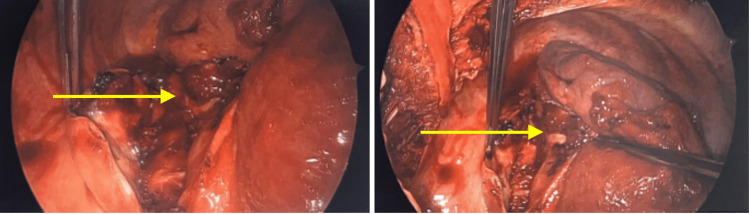
Fibrous necrotic remnants of mediastinal mass around the esophagus (yellow arrow)

Upon the evacuation of the hemothorax and the stabilization of the bleeding, the fibrinous clot (mediastinal mass) was carefully removed from the cystic structure and sent to pathology. The underlying esophagus was identified in this region and appeared to have both acute and chronic inflammation in this region. The cyst wall appeared to be occupying the outer layer of the esophagus and was not removed. A portion of the lateral cyst wall was removed however adjacent to the aorta and sent to the laboratory for pathology analysis. Figure 4 shows the right thoracoscopy revealing a large fibrinous necrotic mass (yellow arrow) measuring 11.5 × 6.5 × 12.5 cm, which was resected post hemothorax evacuation. Figure 5 shows the right thoracic cavity post hemothorax evacuation. Figure 6 shows fibrous necrotic remnants of mediastinal mass around the esophagus.

Histopathologic examination

**Figure 7:**
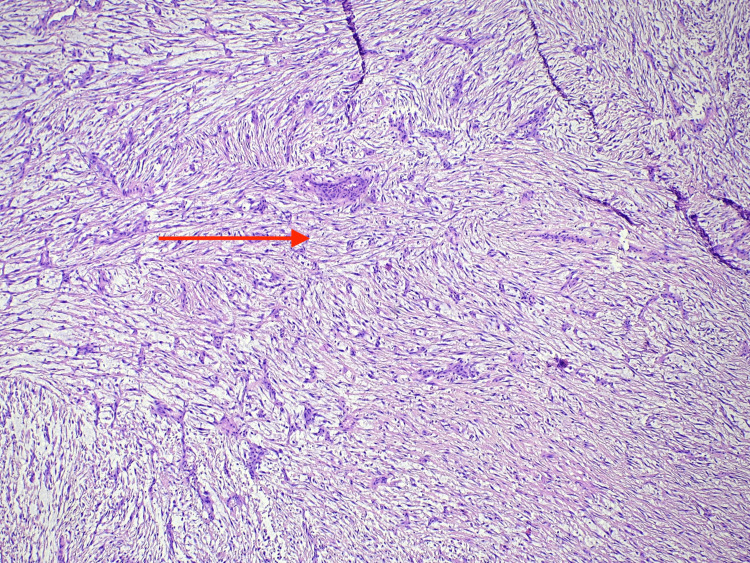
Inflammatory, myofibroblastic, paraesophageal, dense, streaming architecture of the spindle cell myofibroblast (red arrow) sarcoma indicative of malignancy Hematoxylin and eosin (H&E) magnification: 10×

**Figure 8:**
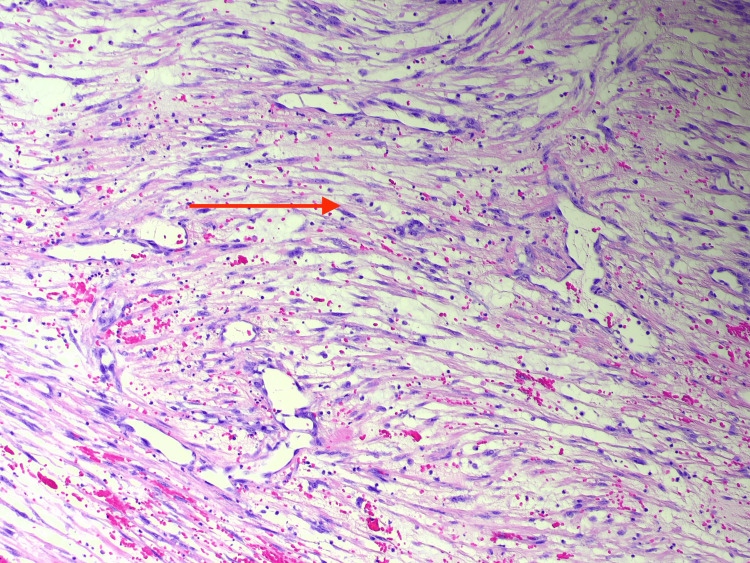
Mediastinal mass histology slide image demonstrating dense capillaries (red arrow), showing the tumor was well perfused with the risk of bleeding and subsequent hemothorax Hematoxylin and eosin (H&E) magnification: 20×

The histological examination revealed distinctive features of the tumor, including elongated nuclei and eosinophilic cytoplasm, composed of spindle-shaped cells. These cells were observed alongside a notable mixed lymphoplasmacytic inflammation. The lesion lacked significant nuclear pleomorphism and showed a brisk mitotic activity. Immunohistochemically, the tumor tested positive for smooth muscle actin (SMA), desmin, caldesmon, and activin receptor-like kinase 1 (ALK1). Conversely, myogenin staining was negative, ultimately confirming the diagnosis of an inflammatory myofibroblastic tumor as shown in Figure 7 and Figure 8.

## Discussion

Inflammatory myofibroblastic tumors (IMTs) were first reported in the late 1930s as lesions of myofibroblastic spindle cells and infiltrates of lymphocytes, plasma cells, and eosinophils [1]. The etiology of IMT diagnosis continues to be controversial. Some argue that these lesions are neoplasms, though they are rarely metastatic [4], while others argue that their occurrences are a result of exposure to a variety of viruses including Epstein-Barr virus and human herpesvirus [5]. These lesions can be found in various tissues of the gastrointestinal tract, the liver retroperitoneum, and the pelvis but rarely in the mediastinum.

The diagnosis of such tumors prior to symptom presentation is very difficult as symptoms may go overlooked by patients as simple discomfort. Our patient presented with progressive dysphagia and shortness of breath along with vital signs of hypertensive emergency. Even when symptoms are reported by patients, approximately 6.3% of IMT cases are discovered via biopsy alone [3]. The lesion in our patient was discovered in the mediastinum post evacuation of a massive hemothorax caused by the ruptured IMT. Most definitive diagnosis continues to be histopathologic and immunohistochemical studies completed after the surgical resection of the tumor. The radical resection of the tumor continues to be the definitive treatment of these findings. Adjuvant therapy administration is not needed once resection is completed.

## Conclusions

In summary, we have presented an exceptionally rare occurrence of massive hemothorax attributed to the rupture of an inflammatory myofibroblastic tumor (IMT) located within the right thoracic cavity. While IMTs are infrequently encountered within the mediastinum of the general population, our report underscores the importance of considering IMT as a differential diagnosis when patients present with progressive dysphagia. This case serves as a poignant reminder of the intricate array of medical conditions that can manifest within the human body and emphasizes the necessity of maintaining a comprehensive differential diagnosis approach, especially in cases with atypical presentations. The management of hemothorax due to IMT rupture necessitates a multidisciplinary approach and collaboration in achieving an accurate diagnosis and implementing a tailored treatment plan.
